# Downregulation of XBP1 protects kidney against ischemia-reperfusion injury via suppressing HRD1-mediated NRF2 ubiquitylation

**DOI:** 10.1038/s41420-021-00425-z

**Published:** 2021-03-02

**Authors:** Ji Zhang, Jiasi Zhang, Haiqiang Ni, Yanfeng Wang, Gaurav Katwal, Yuanyuan Zhao, Kailun Sun, Mengqin Wang, Qingwen Li, Gen Chen, Yun Miao, Nianqiao Gong

**Affiliations:** 1 Institute of Organ Transplantation, Tongji Hospital, Tongji Medical College, Huazhong University of Science and Technology, Key Laboratory of Organ Transplantation of Ministry of Education, National Health Commission and Chinese Academy of Medical Sciences, 430030 Wuhan, Hubei China; 2 Organ Transplant Department, Nanfang Hospital, Southern Medical University, 510515 Guangzhou, China; 3 Institute of Hepatobiliary Diseases, Transplant Center, Hubei Key Laboratory of Medical Technology on Transplantation, Zhongnan Hospital, Wuhan University, 430071 Wuhan, Hubei China; 4 Chitwan Medical College Teaching Hospital, Department of Surgery, Bharatpur, Chitwan, 44200 Nepal; 5 Department of Radiology, Tongji Hospital, Tongji Medical College, Huazhong University of Science and Technology, 430030 Wuhan, Hubei China

**Keywords:** Ubiquitylation, Endoplasmic reticulum, Acute kidney injury, Apoptosis

## Abstract

Ischemia-reperfusion (IR) injury to the renal epithelia is associated with endoplasmic reticulum stress (ERS) and mitochondria dysfunction, which lead to oxidative stress-induced acute kidney injury (AKI). X-box binding protein 1 (XBP1), an ERS response protein, could play a prominent role in IR-induced AKI. In this study, we revealed that XBP1 and its downstream target HRD1 participated in the crosstalk between ERS and mitochondrial dysfunction via regulation of NRF2/HO-1-mediated reactive oxidative stress (ROS) signaling. Mice with reduced expression of XBP1 (heterozygous Xbp1±) were resistant to IR-induced AKI due to the enhanced expression of NRF2/HO-1 and diminished ROS in the kidney. Downregulation of XBP1 in renal epithelial cells resulted in reduced HRD1 expression and increased NRF2/HO-1 function, accompanied with enhanced antioxidant response. Furthermore, HRD1 served as an E3-ligase to facilitate the downregulation of NRF2 through ubiquitination-degradation pathway, and the QSLVPDI motif on NRF2 constituted an active site for its interaction with HRD1. Thus, our findings unveil an important physiological role for XBP1/HRD1 in modulating the antioxidant function of NRF2/HO-1 in the kidney under stress conditions. Molecular therapeutic approaches that target XBP1-HRD1-NRF2 pathway may represent potential effective means to treat renal IR injury.

## Introduction

Renal ischemia-reperfusion injury (IRI) is one of the leading causes of acute kidney injury (AKI), which contributes to high morbidity and mortality rates worldwide^1,2^. Several concomitant factors, such as reactive oxygen species (ROS) production, abnormal protein synthesis and increased cell death, can exacerbate the progression of renal IRI^3–5^. However, substantial improvement of efficacy of renal IRI treatment has yet to be achieved^2,6^, due to limited knowledge of the pathogenesis of IRI. Indeed, a hypothesis has been proposed based on the recent researches on endoplasmic reticulum stress (ERS)^7–9^. Previous studies from our and other groups have suggested that ERS acts as a major contributor to the exacerbation of IRI^10–13^. Thus, molecular remodeling of ERS pathway may serve as a new therapeutic strategy for renal IRI^10,14,15^.

Cellular adaptation to ERS is achieved by the activation of unfolded protein response (UPR), which involves three principal ERS sensors, such as inositol-requiring enzyme 1α (IRE1α), protein kinase R-like ER kinase (PERK), and activating transcription factor 6 (ATF6)^16–18^. Notably, the most highly conserved branch of UPR is IRE1α-X-box-binding protein 1 (XBP1) signaling^19–21^. IRE1α autophosphorylation activates the endoribonuclease activity to splice *Xbp1* mRNA (unspliced *Xbp1* or *Xbp1u*, a nonactive form of ERS), thereby changing the reading frame of *Xbp1* to encode a potent transcription factor (termed as XBP1s, the active form of ERS)^22^. XBP1s plays a crucial role in cell survival and death by upregulating UPR-related genes involved in the entrance of proteins into endoplasmic reticulum^23^. Apart from its multiple roles in neurodegenerative and metabolic diseases^20^, IRE1α-XBP1 pathway has been reported to cause AKI in patients receiving cardiac surgery^24^. Furthermore, XBP1s also plays a role in sepsis-induced AKI and inflammation^25^, and inhibition of ERS (including XBP1) protects kidney against rhabdomyolysis-induced AKI^26^. However, sepsis-induced AKI (LPS-related) has been driven by markedly different molecular pathologic characteristics from IR-induced AKI^25^, and the association between XBP1 and significant IR-induced AKI still remains to be elucidated^25,27^.

Hypoxia and reoxygenation can result in a massive ROS release^28^, which promotes mitochondrial damage and participates in the crosstalk between ERS and mitochondrial dysfunction^29^, leading to tubular cell damage and renal injury^30^. Apart from regulating the main effector molecules as reported in our previous work^31^, ROS attenuation remains an effective option to protect kidney against IRI via nuclear factor erythroid 2-related factor 2 (NRF2) and heme oxygenase-1 (HO-1)^32^. The transcription factor NRF2 is responsible for the regulation of various antioxidant and antiapoptotic genes, primarily in response to oxidative stress, and thus increases the levels of endogenous antioxidants and ameliorates cell apoptosis^33^. HO-1 is modulated by NRF2 activation, which acts and functions as a rate-limiting enzyme of heme catabolism for attenuating ROS production^34,35^. Hitherto, the current knowledge on NRF2 modulation remains grossly inadequate^36^. It is believed that NRF2 can be dual-regulated by Kelch-like ECH-associated protein 1 (Keap1) and glycogen synthase kinase 3 beta (GSK-3β), in response to oxidative stress^37–39^. As a key downstream component of XBP1, HRD1 acts as the core structural component of a large ER membrane-embedded protein complex^40^, and is initially characterized as an E3 ubiquitin ligase that coordinates the destruction of folding-defective proteins in the early secretory pathway to prevent ERS-associated degradation^41^. A recent study on liver cirrhosis has found that HRD1 can suppress NRF2-mediated cellular protection^40^. However, no information on the specific motifs of NRF2 that interact with HRD1 has been reported. Therefore, whether and how XBP1-HRD1 and NRF2-HO-1 exhibit intrinsic relevance and manipulate the crosstalk between ERS and mitochondrial dysfunction in kidney await further clarification.

In this study, we investigated the roles of XBP1 on IR-induced AKI and revealed the mechanisms of NRF2-HO-1 mediated regulation of XBP1-HRD1. The findings provide novel insights into the therapeutic implications of the XBP1-HRD1-NRF2 pathway for treating AKI.

## Results

### XBP1 is involved in the crosstalk between mitochondrial damage and ERS in renal IRI

**Fig. 1:**
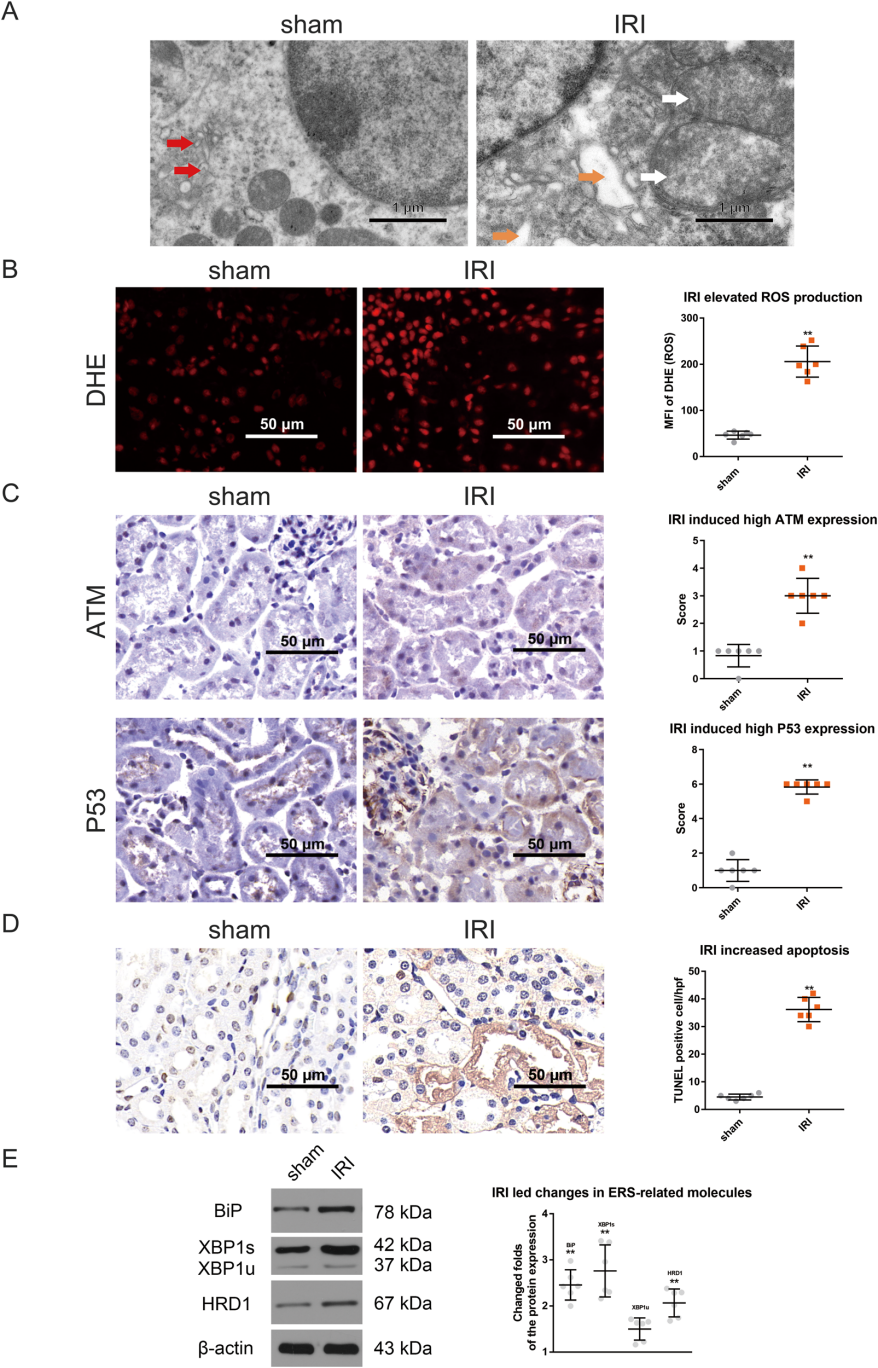
XBP1 is involved in the crosstalk between mitochondrial damage and ERS in renal IRI. **A** Mitochondrial damage and severe ERS occurred simultaneously. TEM analysis revealed mitochondrial swelling, mitochondrial cristae and membrane disappearance, as well as the obvious swelling and vacuolization of ER and detachment of ribosomes from rough ER in IRI group. Normal ER is denoted by red arrows, swelling ER is represented by orange arrows, and swelling mitochondria is indicated by white arrows. Scale bar = 1 μm; ×7000 magnification. **B** IRI significantly elevated the levels of ROS in IRI group compared to sham group. DHE staining; ×400 magnification; scale bar = 50 μm. **P < 0.01 vs. sham group (*n* = 6). **C** The expression of ATM and P53 were raised in IRI group than in sham group. Immunohistochemistry; ×400 magnification; scale bar = 50 μm. **P < 0.01 vs. sham group (*n* = 6). **D** The number of apoptotic cells was higher in IRI group than in sham group. TUNEL staining; ×400 magnification; scale bar = 50 μm. **P < 0.01 vs. sham group (*n* = 6). **E** The fold changes in the expression of ERS-related molecules in the renal tissues after IRI. The expression levels of BiP, XBP1u, XBP1s, and HRD1 were measured by western blotting. Compared to sham group, the expression levels of ERS-related molecules were all increased in IRI group, especially XBP1s. **P < 0.01 vs. sham group (*n* = 6).

After 24 h of IR exposure, the ultrastructural changes in renal tissues were analyzed by transmission electron microscopy (TEM). Both mitochondrial damage (mitochondrial swelling, mitochondrial cristae and membrane disappearance) and ERS (obvious swelling and vacuolization, and ribosome detachment from rough ER) were observed (Fig. 1A). The level of ROS, which reflects mitochondrial damage, was significantly elevated in IRI group compared to sham group (205.75 ± 33.72 vs. 46.40 ± 8.53, respectively; *P* < 0.01; Fig. 1B). The expression of ATM and P53 were significantly enhanced in IRI group than in sham group (3.00 ± 0.63 vs. 0.83 ± 0.40, 5.83 ± 0.40 vs. 1.00 ± 0.63, respectively; *P* < 0.01; Fig. 1D). The number of apoptotic cells was significantly higher in IRI group than in sham group (36.17 ± 4.40 vs. 4.50 ± 1.05, respectively; *P* < 0.01; Fig. 1D). Latterly, the expression levels of ERS-related molecules (BiP, XBP1u, XBP1s, and downstream HRD1) were determined by western blotting. Their relative fold changes in IRI kidneys were significantly increased compared to those in normal kidneys (2.45 ± 0.32, 2.75 ± 0.56, 1.48 ± 0.24, and 2.07 ± 0.30, respectively; *P* < 0.01; Fig. 1E). Among which, the upregulation of XBP1s was prominent. Collectively, these findings indicate the presence of crosstalk between mitochondrial damage and severe ERS in renal IRI, and the prominent high expression levels of XBP1s suggest that XBP1 may play a unique role in the crosstalk between mitochondrial damage and ERS during renal IRI.

### Mice with *Xbp1* downregulation are resistant to IR-induced AKI due to the enhanced expression of NRF2/HO-1 and diminished ROS in kidneys

**Fig. 2:**
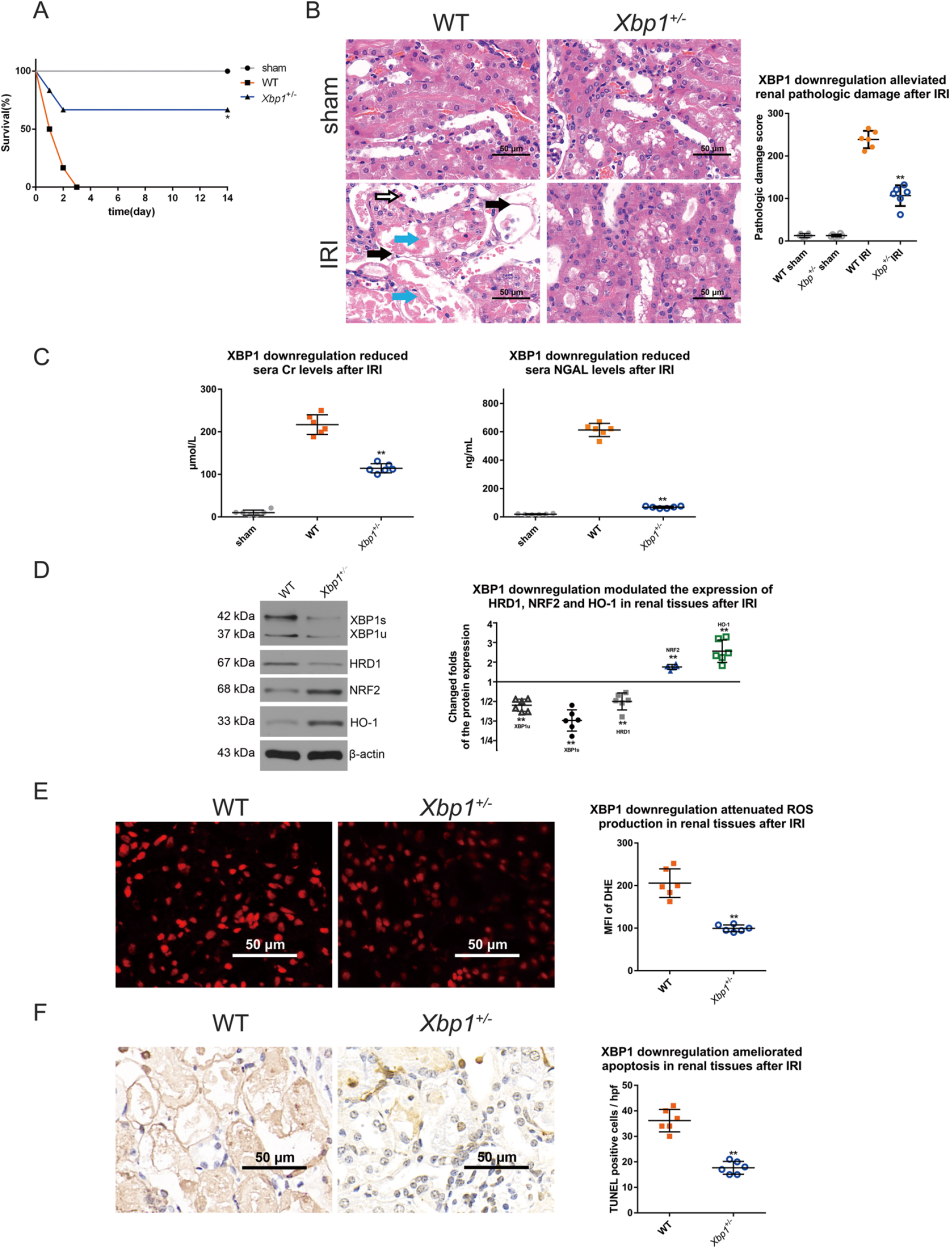
Mice with reduced *Xbp1* are resistant to IRI-induced AKI due to the enhanced expression of NRF2/HO-1 and diminished ROS in kidneys. **A** XBP1 downregulation significantly prolonged the survival of *Xbp1*^+/−^ mice. *P < 0.05 vs. IRI group (*n* = 6). **B** XBP1 downregulation significantly alleviated acute renal damage and decreased pathological damage scores. H&E staining; scale bar = 50 μm, ×400 magnification. **P < 0.01 vs. IRI group (*n* = 6). Tubular necrosis is denoted by light blue arrows, sloughing of epithelial cells are represented by white arrows, and tubular basement membrane damage are indicated by black arrows. H&E staining; ×400 magnification; scale bar = 50 μm. **P < 0.01 vs. the sham group (*n* = 6). **C** XBP1 downregulation significantly reduced the sera levels of Cr and NGAL. **P < 0.01 vs. IRI group (*n* = 6). **D** XB*P*1 downregulation significantly decreased the expression levels of XBP1 and HRD1, while increased the expression levels of NRF2 and HO-1. Western blot; **P < 0.01 vs. IRI group (*n* = 6). **E** IR-induced ROS production was significantly attenuated in the kidneys of *Xbp1*^+/−^ mice, as shown by both MFI of DHE. Scale bar = 50 μm; ×400 magnification. **P < 0.01 vs. IRI group (*n* = 6). **F** XBP1 downregulation significantly reduced the number of apoptotic cells. TUNEL assay; scale bar = 50 μm, ×400 magnification. **P < 0.01 vs. IRI group (*n* = 6).

We next tested the potential role of XBP1 in the mouse model of renal IRI. Twenty-four hours after IR, XBP1 downregulation significantly prolonged the survival of heterozygous *Xbp1*^+/−^ mice, with a higher rate of 66.66% compared to wild-type (WT) mice (*P* < 0.05; Fig. 2A). Pathological examination revealed the severe diffuse acute tubular necrosis, sloughing of epithelial cells and tubular basement membrane damage in WT renal tissue exposed to IR. The pathological score (106.83 ± 24.39) of *Xbp1*^+/−^ mice was significantly lower than that (238.83 ± 20.38) of WT mice (*P* < 0.01; Fig. 2B), indicating that XBP1 downregulation can alleviate pathological damage in kidneys. Moreover, the function of kidney was determined by assessing the serum levels of Cr and NGAL 24 h after IR. The serum levels of Cr were 10.15 ± 6.10, 216.92 ± 23.13, and 114.33 ± 10.75 μmoI/L in sham, WT and *Xbp1*^+/−^ groups, respectively (*P* < 0.01; Fig. 2C), while those of NGAL were 19.42 ± 3.66, 613.01 ± 46.51, and 68.56 ± 7.44 ng/mL in the three groups, respectively (*P* < 0.01; Fig. 2C), suggesting Xbp± kidneys were more resistant to IR-induced dysfunction than those of WT mice.

The kidneys of WT and *Xbp1*^+/−^ mice were harvested for Western blot analysis, in order to determine the expression levels of XBP1/HRD1 and NRF2/HO-1 proteins. Compared to WT controls, *Xbp1*^+/−^ renal tissues exhibited significant decreases in the expression levels of XBP1 and HRD1, while significant increases in those of NRF2 and HO-1 (Fig. 2D). In addition, the ROS level in the kidneys of *Xbp1*^+/−^ mice was markedly reduced compared to WT controls (99.62 ± 8.02 vs. 205.75 ± 33.72, respectively; *P* < 0.01; Fig. 2E). These data show that XBP1 downregulation effectively modulates the expression of XBP1, HRD1, NRF2, and HO-1, and consequently lead to ROS suppression. Thereafter, we verified whether the downregulated expression of XBP1 can attenuate cell apoptosis. As shown in Fig. 2F, the number of apoptotic cells was remarkably lower (*P* < 0.01) in *Xbp1*^+/−^ group (17.67 ± 2.50) than in WT group (36.17 ± 4.40). Taken together, we found that downregulation of XBP1 protected against renal IRI via inhibiting ROS formation and promoting cell survival. The molecular mechanisms underlying downregulation of XBP1 might include downregulation of a known target of XBP1, HRD1 and upregulation of NRF2 and HO-1.

### XBP1 downregulation protects tubular cells against hypoxia/reoxygenation (H/R) damage via control of HRD1, NRF2, and HO-1

**Fig. 3:**
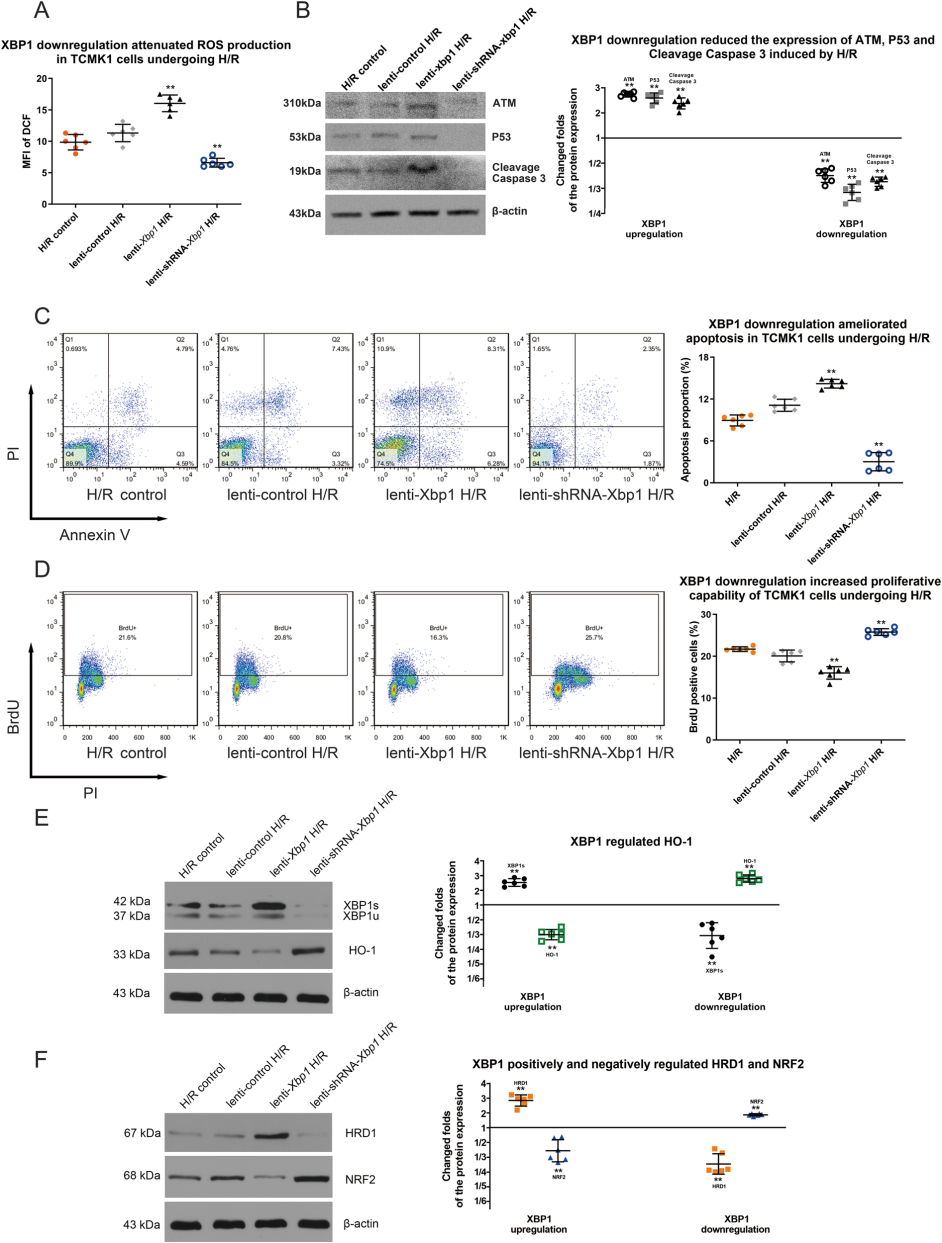
XBP1 downregulation protects TCMK-1 cells against H/R via regulation of HRD1, NRF2, and HO-1. **A** The level of ROS was attenuated by lenti-shRNA-*Xbp1* transduction in H/R-exposed TCMK-1 cells. DCFH-DA assay; **P < 0.01 vs. lenti-control H/R group at each time point (*n* = 6). **B** P53, ATM, and cleavage caspase 3 expression were ascended by lenti-*Xbp1* transduction and descended by lenti-shRNA-*Xbp1* transduction in H/R-exposed TCMK-1 cells. Western blot; **P < 0.01 vs. lenti-control H/R group (*n* = 6). **C** The apoptotic proportion of H/R-exposed TCMK-1 cells was decreased by lenti-shRNA-*Xbp1* transduction, as revealed by flow cytometry. **P < 0.01 vs. lenti-control H/R group at each time point (*n* = 6). **D** The BrdU incorporation (proliferative capability) of H/R-exposed TCMK-1 was enhanced by lenti-shRNA-*Xbp1* transduction. *P < 0.05 vs. lenti-control H/R group at each time point (*n* = 6). **E** HO-1 expression was negatively modulated by XBP1 expression following lenti-*Xbp1* and lenti-shRNA-*Xbp1* transduction. Western blot; **P < 0.01 vs. lenti-control H/R group (*n* = 6). **F** XBP1 positively and negatively regulated HRD1 a*n*d NR**F**2, respectively. *P < 0.05, **P < 0.01 vs. lenti-control H/R group (*n* = 6).

Next, we sought to confirm the molecular actions of XBP1 in IRI. First, we manipulated expression of XBP1 with both lentivirus mediated overexpression and siRNA knockdown in mouse kidney epithelial cell line (TCMK-1) (Fig. S2, S3). Then, different cell lines were subjected to H/R injury. As shown in Fig. 3A, the levels (MFI of DCF) of ROS were reduced in XBP1 knockdown cells and increased in XBP1 overexpression cells as compared to control cells (11.32 ± 1.38 in lenti-control H/R, 16.03 ± 1.32 lenti-Xbp1 and 6.59 ± 0.71 in lenti-shRNA-Xbp1). Meanwhile, the expression of P53, ATM, and cleavage caspase 3 were descended in XBP1 knockdown cells and enhanced in XBP1 overexpression cells as compared to control cells (Fig. 3B). Similarly, flow cytometric analysis revealed that the apoptotic cells were small reduced in XBP1 knockdown cells as compared to that in control cells (11.12 ± 0.86 % in in lenti-control H/R and 3.02 ± 1.30% in lenti-shRNA-Xbp1, Fig. 3C). Additionally, the proliferative capacity of H/R-exposed TCMK-1 cells was significantly enhanced in lenti-shRNA-Xbp1 group (Fig. 3D). These findings demonstrated that XBP1 downregulation may ameliorate H/R injury in vitro.

To further explore the molecular action of XBP1 in response to H/R injury, the expression levels of HO-1 were examined, and the results showed that HO-1 was significantly downregulated in lenti-Xbp1 and upregulated in lenti-shRNA-Xbp1 cells as compared to that in control cells (*P* < 0.01; Fig. 3E), suggesting that XBP1 negatively regulates HO-1 expression. Furthermore, western blot analysis revealed that XBP1 downregulation suppressed HRD1 expression and induced NRF2 expression, while XBP1 upregulation increased HRD1 expression and decreased NRF2 expression (Fig. 3F). These findings show that XBP1 positively and negatively regulates HRD1 and NRF2/HO-1, respectively.

### HRD1 modulates NRF2 through ubiquitination degradation

**Fig. 4:**
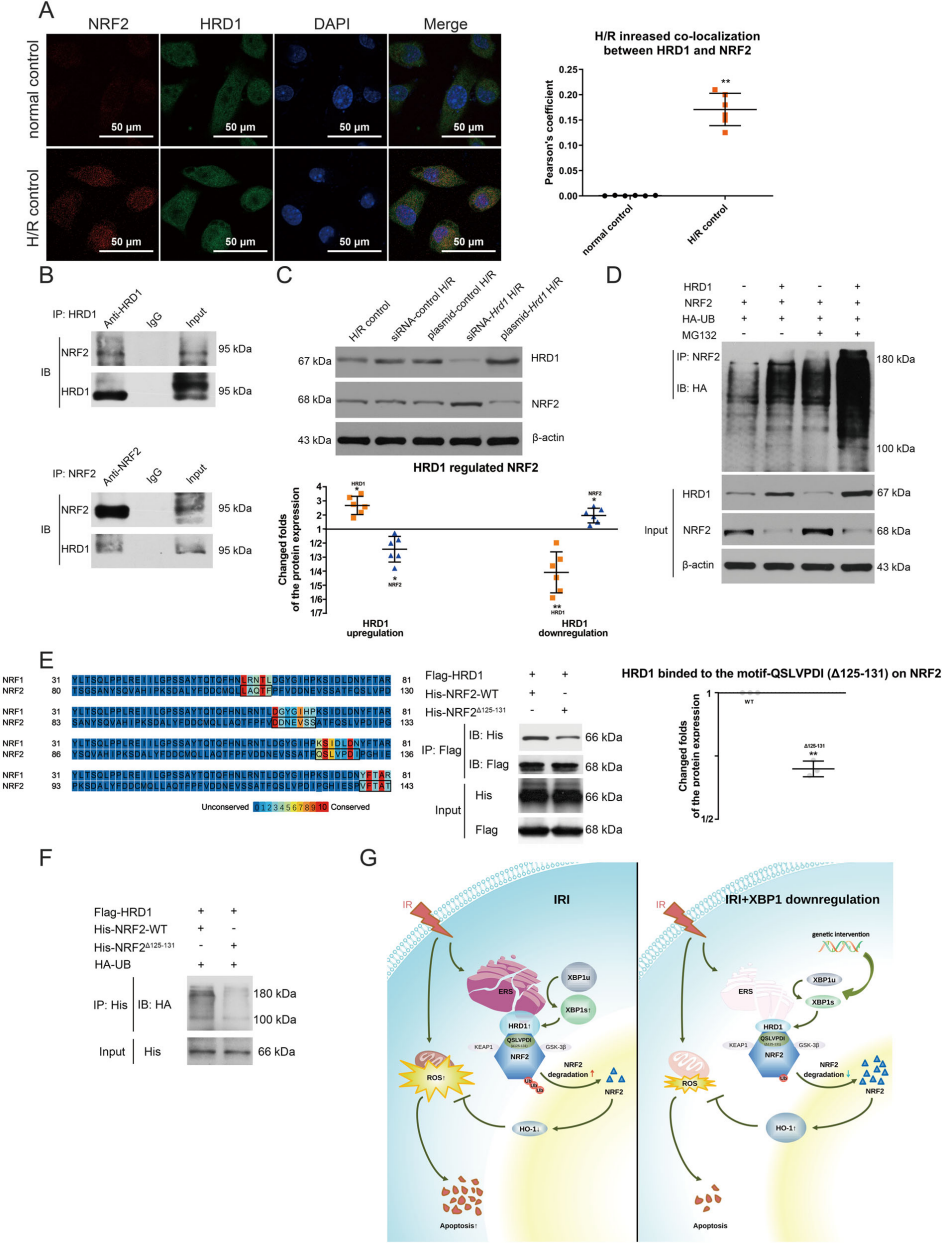
HRD1 modulates NRF2 through ubiquitination degradation. **A** Colocalization of HRD1 and NRF2 in H/R-exposed TCMK-1 cells. Confocal microscopy analysis; Pearson’s correlation coefficient; scale bar = 50 μm; ×600 magnification. **P < 0.01 vs. normal control group (*n* = 6). **B** HRD1 and NRF2 coexisted in the precipitated complexes of H/R-exposed TCMK-1 cells. IP immunoprecipitation, IB immunoblot,. **C** Silencing of HRD1 led to an increase in NRF2 level, while overexpression of HRD1 caused a reduction in NRF2 level. **D** HRD1 overexpression promoted NRF2 ubiquitylation and degradation, and subsequently reduced NRF2 level in HEK-293T cells. **E** The QSLVPDI motif on NRF2 constituted an active site for its interaction with HRD1. **F** The deletion of QSLVPDI amino acids sequence (Δ125–131) remarkably decrease ubiquitylation of NRF2 mediated by HRD1. **G** Graphical illustration of the working mechanism: XBP1/HRD1 is involved in IR-induced AKI through the regulation of NRF2/HO-1-mediated ROS signaling.

As it was shown that HRD1 served as an E3 ubiquitin ligase for NRF2 in liver cirrhosis^40^, we performed the following experiments to test whether HRD1 could regulate NRF2 in the setting of renal IR injury as well. First, confocal images showed colocalization between HRD1 and NRF2 was increased in H/R-exposed TCMK-1 cells compared to normal cells, with the Pearson’s coefficient values of (1708.00 ± 130.70) × 10^−4^ and (5.55 ± 0.53) × 10^−4^, respectively, (*P* < 0.01; Fig. 4A), indicating an enhanced endogenous interaction between HRD1 and NRF2 after H/R exposure. Next, co-immunoprecipitation analysis demonstrated that HRD1 and NRF2 apparently coexisted in the immune-precipitated complexes in H/R-exposed TCMK-1 cells (Fig. 4B). Moreover, neither upregulation nor downregulation of HRD1 could alter XBP1 expression, while HRD1 negatively regulated NRF2 expression (Fig. 4C). In contrast, alteration of NRF2 had no effects on the expression of XBP1 nor HRD1 (Fig. S4). Furthermore, ubiquitylation assay was used to determine the ubiquitylation of NRF2 by HRD1 in HEK-293T cells. Overexpression of HRD1 promoted NRF2 ubiquitylation, thus reducing the level of NRF2 via degradation (Fig. 4D). Taken together, HRD1 is an E3 ubiquitin ligase of NRF2 in IR conditions.

Recent data have suggested a direct interaction between NRF2 Neh4-5 domains and HRD1^40^. Through a bioinformatic approach, we identified four conserved sequences (Δ107–111, Δ115–121, Δ125–131, and Δ139–143) in Neh4-5 domains of NRF2 that potentially bind with HRD1 (Fig. 4E). To further verify whether these sequences can act as the motifs of NRF2 that interact with HRD1, an in vitro binding assay was performed by using HA-tagged ubiquitin proteins, Flag-tagged HRD1 proteins and His-tagged NRF2 proteins with deletion mutants in Δ107–111, Δ115–121, Δ125–131, or Δ139–143 domain and immunoprecipitated from HEK-293T cells. The results showed that the deletion of QSLVPDI amino acids sequence (Δ125–131) remarkably diminished the interaction between NRF2 and HRD1 and ubiquitylation of NRF2, meaning HRD1 binded to the motif-QSLVPDI (Δ125–131) on NRF2 to ubiquitylate NRF2 (Fig. 4E, F). In addition, motif-QSLVPDI (Δ125–131) was proven to have no effect on Keap1 and GSK-3β (Fig. S5). In summary, our study suggested that renal IRI could induce expression of XBP1 and its target HRD1, the latter serving as an E3-ligase of NRF2 to inhibit NRF2/HO-1 signaling in mitochondria (as illustrated in Fig. 4G).

## Discussion

In the kidneys undergoing IR-induced AKI, at ultrastructural level, TEM observed mitochondrial damage and severe ERS, supporting the co-presence of mitochondrial dysfunction and ERS after renal IRI. IRI involves a complex pathological process where the tissue injury is initiated by H/R, leading to damaged electron transport chain and high ROS production in mitochondria^42^. With recovery of oxidation, excessive ROS can be generated from the oxidative damage^43^. We demonstrated a large amount of ROS (denoting the severity of mitochondrial damage) and elevated apoptosis following IRI Based on the current knowledge that ERS can crosstalk with ROS-induced mitochondrial damage in an infection model^29^, XBP1s pathway is associated with mitochondrial function^44,45^, and N-acetylcysteine abrogates mitochondrial ROS through ERS and thus alleviates myocardial apoptosis^46^, our findings indicate a potential role of XBP1 in crosstalk of ERS and mitochondrial damage in renal IRI. Indeed, modulation of XBP1 led to complex alteration of both the ERS signaling factor (HRD1) and mitochondrial damage factors (NRF2/HO-1).

The protective effects of XBP1 downregulation on kidney against IRI were subsequently tested. Due to mouse kidney’s high tolerability on IR, mild IR injury by the reported 20-min ischemia and 24-h reperfusion failed to affect XBP1 expression in mice kidney^25^, and exerted no significant impact on renal function and survival. Therefore, we used a severe IR injury model (45-min ischemia and 24-h reperfusion) to archive clinical significance. Though the previous study has shown that XBP1s hyperactivity is detrimental to kidney^25^, XBP1 is necessary for survival. Complete knockout of XBP1 resulted in embryonic lethality^47,48^, and deletion of an XBP1 allele in mice led to enhanced ERS resulting in systemic insulin resistance and insulin receptor substrate-1 degradation^47,49,50^. Therefore, in this work, heterozygous *Xbp1*^+/−^ mice were used as an animal model for XBP1 downregulation. Following IRI, the survival rate of heterozygous *Xbp1*^+/−^ mice was significantly increased compared to WT mice. The IR-exacerbated pathological damages and kidney function (sera Cr and NGAL) had shown significant improvement in heterozygous *Xbp1*^+/−^ mice. The underlying molecular events were further clarified. XBP1 expression was shown to be downregulated in the renal tissues of *Xbp1*^+/−^ mice after IRI. Consequently, XBP1 downregulation decreased HRD1 expression and increased the expression of NRF2 and HO-1. XBP1 has been recently reported positive regulation on NRF2 expression in retinal pigment epithelium^51^. As results, both ROS levels and apoptosis in the renal tissues were attenuated. In addition, researchers also observed the effects of XBP1 downregulation on the innate and adaptive immune systems during renal IRI process^1,52,53^, which await further investigation into the underlying mechanism. Collectively, mice with Xbp1 downregulation are resistant to IR-induced AKI due to the enhanced expression of NRF2/HO-1 and diminished ROS in kidneys.

To precisely describe the mechanism of XBP1 on renal IRI, TCMK-1 cells were used. XBP1 is recognized as a stress-inducible transcriptional activator of ERS^54–56^. Our study demonstrated that lenti-*Xbp1* transduction effectively upregulated the expression of XBP1s (the active form), while lenti-shRNA-*Xbp1* transduction remarkably downregulated its expression. Moreover, we found that XBP1 upregulation elevated ROS levels, increased apoptosis, and reduced proliferation in H/R-exposed tubular cells, while XBP1 downregulation exhibited the opposite effects. Therefore, modulation of XBP1 can regulate ROS production and apoptosis of tubular cells undergoing H/R.

It is well accepted that HO-1 is modulated by NRF2, and HRD1 represents the key downstream molecules of XBP1^57^. Our studies suggested XBP1 could regulate NRF2/HO-1 signaling via regulating HRD1 expression. In turn, mitochondrial ROS formation was altered by XBP1.

Next, we sought to determine the mechanisms underlying HRD1-mediated regulation of NRF2. First, a strong colocalization between HRD1 and NRF2 was detected in the cytoplasm of H/R-exposed tubular cells. Second, co-immunoprecipitation analysis revealed a molecular conjunction between HRD1 and NRF2. As an E3 ubiquitin ligase^58^, HRD1 may degrade NRF2 by identifying protein substrates and facilitating or directly catalyzing the binding of ubiquitin after H/R exposure. The results of ubiquitylation assay showed that HRD1 overexpression promoted NRF2 ubiquitylation and reduced the expression level of NRF2 protein. Furthermore, HRD1 was demonstrated to bind with the motif-QSLVPDI (Δ125–131), but not other motifs (Fig. S6), on both NRF2 and ubiquitylated NRF2.

In summary, our study unveils that ERS signaling axis, XBP1/HRD1, participates in the crosstalk between ERS and mitochondrial dysfunction through control of NRF2/HO-1-mediated ROS signaling. IR increases XBP1-HRD1 expression and promotes interaction between HRD1 and NRF2, which in turn induce ubiquitylation and degradation of NRF2. The interaction domain within NRF2 is the motif-QSLVPDI (Δ125–131). Downregulation of NRF2 induces ROS production, impairs kidney function and shortens animal survival. Thus, downregulation of XBP1 can effectively protect kidney against IRI, which may be of great importance in the development of novel XBP1-based therapeutic strategies for kidney protection against IRI.

## Materials and methods

### Experimental mice

Wild-type (WT) C57BL/6 mice were purchased from Beijing Vital River Laboratory Animal Technology Co., Ltd. (China). Heterozygous *Xbp1*^+/−^ mice (C57BL/6NTac-Xbp1 < tm1a(EUCOMM)Wtsi > /IcsOrl mice) were obtained from the European Mouse Mutant Cell Repository (Phil Avner, Paris, France) via Beijing Vital River Laboratory Animal Technology Co., Ltd. (China). The *Xbp1* allele contained a trapping cassette SA-βgeo-pA (splice acceptor-beta-geo-polyA) flanked by flippase (Flp) recombination target (FRT) site upstream of exon. This resulted in the truncation of endogenous transcript, and thus forming a constitutive null mutation to knockdown XBP1 expression. The FRT-flanked region of heterozygous *Xbp1*^+/−^ mice was detected by genotyping using PCR assay (Fig. S1).

All animals were housed in specific pathogen-free facilities and maintained under controlled conditions (22 °C, 55% humidity and 12 h day/night cycle) at Huazhong University of Science and Technology animal center. All animal experiments were approved by the Institutional Animal Care and Use Committee at Tongji Hospital, and carried out in accordance with the Experimental Animal Management Ordinance (National Science and Technology Committee of China).

### Renal IRI model

Renal IRI was induced on female mice aged 8–10 weeks as described previously^59^. The left renal pedicle was occluded for 45 min, followed by reperfusion. After that, right kidney nephrectomy was performed. The mice were randomized into the following groups: (1) sham group: mice received sham surgery; (2) WT group: mice with left kidney IRI; and (3) *Xbp1*^+/−^ group: *Xbp1*^+/−^ mice with left kidney IRI. Six animals in each group were studied for their survival rates, while three animals were selected for mechanistic study for six times. The animals were sacrificed 24 h after reperfusion, in order to collect serum and left kidney samples.

### TEM

The left kidneys in sham and IRI groups were prepared for semi-thin sections (0.5–1 mm in thickness). The sections were stained with methylene blue and examined under transmission electron microscope (Hitachi, Tokyo, Japan).

### Measurement of ROS levels in renal tissues

Left kidneys were dissected 24 h after IRI, rapidly frozen in liquid nitrogen, cut at 2 μm in a cryostat (CryoStar NX50, Thermo Fisher Scientific, Inc., Waltham, MA, USA), and stained with dihydroethidium (DHE; Sigma–Aldrich; Merck KGaA, Darmstadt, Germany). The stained sections were observed under a fluorescence microscope (ECLIPSE C1, NIKON, Tokyo, Japan). ROS production in renal tissues was evaluated by determining the mean fluorescence intensities (MFI) of DHE.

### Immunohistochemistry

Immunohistochemistry of ATM and P53 was performed on formalin-fixed and paraffin-embedded sections according to the previously described procedure^60^. Images were captured under the Olympus BX-51 light microscope. The staining intensity and proportion of positive cells were calculated as previously described^61^. The intensity was scored as follows, 0 = negative, 1 = weak, 2 = moderate, 3 = strong. Proportion scores are points assigned based on the percentage of positive cells as follows: <10% for 0 point, 10–25% for 1 point. 26–50% for 2 point, 51–75% for 3 point, and >75% for 4 point. A total score was obtained by multiplying intensity score × proportion score. The total score ranged from 0 to 12.

### Terminal dUTP nick end labeling (TUNEL)

Cell apoptosis in paraffin sections was evaluated by TUNEL assay using the In Situ Cell Death Detection Kit, POD (Roche, Basel, Switzerland) according to the manufacturer’s instructions. Sections of formalin-fixed and paraffin-embedded kidney tissue were dewaxed by washing in xylene and rehydrated through a gradual series of ethanol and distilled water. Proteinase K-permeabilized sections were subjected to enzymatic in situ labeling of DNA strand breaks using TUNEL technique as it is indicated in manufacturer instruction. Apoptosis was assessed using the Olympus BX-51 light microscope, six images were captured in randomly selected fields from each section and the number of TUNEL-positive cells were counted.

### Histology staining

Left kidneys were dissected from mice 24 h after IRI and fixed in 10% formalin. The formalin-fixed kidneys were embedded in paraffin and sectioned at 4 μm thickness. The sections were then subjected to hematoxylin and eosin (H&E) staining. Semi-quantitative analysis was performed as previously described^62^.

Random cortical fields were observed using a ×20 objective. A graticule grid (25 squares) was used to determine the number of line intersects involving tubular profiles. One hundred intersections were examined for each kidney, and a score from 0 to 3 was given for each tubular profile involving an intersection: 0 = normal histology; 1 = tubular cell swelling, brush border loss, nuclear con-densation, with up to one third of the tubular profile showing nuclear loss; 2 = same as for score 1, but greater than one third and less than two thirds of the tubular profile show nuclear loss; and 3 = greater than two thirds of the tubular profile showing nuclear loss. The total score for each kidney was calculated by the addition of all 100 scores with a maximum score of 300.

Images were recorded under a light microscope (Olympus BX-51, Tokyo, Japan), and then analyzed using ImageJ software.

### Sera measurement

Serum creatinine (Cr) concentrations were measured at the clinical laboratory of Tongji Hospital (Wuhan, China). The concentrations of neutrophil gelatinase associated lipocalin (NGAL) were determined using LEGEND MAX™ Mouse NGAL ELISA kit (Biolegend Inc., San Diego, CA, USA).

### Cells

The mouse kidney epithelial cell line (TCMK-1) and human embryonic kidney cells (HEK-293T) were purchased from ATCC (Manassas, VA, USA). TCMK-1 cells were cultured in RPMI 1640 (HyClone; GE Healthcare, Logan, UT, USA) supplemented with 10% heat-inactivated fetal bovine serum (FBS; Gibco, Thermo Fisher Scientific, Inc., Waltham, MA, USA). HEK-293T cells were cultured in high glucose Dulbecco’s modified eagle medium (DMEM; HyClone; GE Healthcare, Logan, UT, USA) supplemented with 10% FBS. The cultured cells were maintained at 37 °C in a humidified incubator containing 5% CO_2_.

### Plasmids, small interfering RNA (siRNA), and lentiviruses

Plasmids expressing mouse *Hrd1*, human *Flag*-*HRD1*, human *Flag*-*KEAP1*, human *Flag*-*GSK-3β*, human *His-NRF2*, and *HA*-ubiquitin (human) as well as control plasmid were purchased from Vigene Bioscience Co., Ltd. (Rockville, MD, USA).

The conserved sequences between the Neh4-5 domain of Nrf2 and the residues 31–81 of NRF1 were predicated using PRALINE (http://www.ibi.vu.nl/programs/pralinewww/). The deletions of Δ107–111, Δ115–121, Δ125–131, and Δ139–143 were constructed within pcDNA3.1/ His-NRF2 by site-directed mutagenesis (SDM) using the primers listed in Table S1.

Mouse *Hrd1* siRNA and control siRNA were purchased from Guangzhou Ribobio Co., Ltd. (Guangzhou, China). Mouse *Xbp1* lentivirus, mouse *Xbp1* shRNA lentivirus and control lentivirus were purchased from Hanbio Co., Ltd. (Shanghai, China). The sequences of siRNA and shRNA are presented in Table S2.

Transfection of plasmid and siRNA was performed using Lipofectamine 2000 Transfection Reagent (Invitrogen; Thermo Fisher Scientific, Inc., Waltham, MA, USA) according to the manufacturer’s instructions. Transduction of lentiviruses into TCMK-1 cells was carried out as described previously^63^.

### Inhibitor and activator of NRF2

To downregulate NRF2 expression, ML385 (Sigma–Aldrich; Merck KGaA, Darmstadt, Germany), a specific NRF2 inhibitor, was added into the culture medium at a concentration of 5 μM, followed by incubation for 48 h. Meanwhile, CDDO-Me (Sigma–Aldrich; Merck KGaA, Darmstadt, Germany), a specific NRF2 activator, was used to upregulate NRF2 expression by incubating for 48 h at a concentration of 0.5 μM.

### Cell hypoxia/reoxygenation (H/R) injury

For hypoxic induction, TCMK-1 and HEK-293T cells were incubated at 37 °C in a humidified atmosphere containing 5% CO_2_, 1% O_2_ and 94% N_2_ for 24 h. Then, the cells were reoxygenated under normal conditions for 2 h. The details of TCMK-1 cell grouping are summarized in Table S3.

### Flow cytometry

Cell apoptosis was evaluated after labeling with propidium iodide (10 μL; Biolegend Inc., San Diego, CA, USA) and Annexin V (5 μL; Biolegend Inc., San Diego, CA, USA). The production of ROS was measured using the Total ROS Assay Kit (Invitrogen; Thermo Fisher Scientific, Inc., Waltham, MA, USA). Cell proliferation was evaluated by BrdU Cell Proliferation Assay Kit (MultiSciences, Hangzhou, China).

### Immunofluorescence

TCMK-1 cell culture slides and renal tissue sections were incubated with primary antibodies at 4 °C overnight. Secondary antibodies were then added and incubated at 37 °C for 50 min. Cell nuclei were stained with 4’,6-diamidino-2-phenylindole (DAPI). The details of antibodies are provided in Table S4. Images were obtained using a fluorescence microscope (ECLIPSE C1, NIKON, Tokyo, Japan). A confocal laser scanning microscope (FV1000, Olympus, Tokyo, Japan) was used to examine the colocalization between HRD1 and NRF2 molecules. Images processing and analysis were performed using with ImageJ software.

### Quantitative real-time reverse transcriptase polymerase chain reaction (qRT-PCR)

Total RNA was isolated from TCMK-1 cells and kidney samples using TRIzol® (Invitrogen; Thermo Fisher Scientific, Inc., Waltham, MA, USA). Total RNA was reverse-transcribed into cDNA using the PrimeScript™ RT reagent kit (Perfect Real Time; Takara Bio Inc., Otsu, Shiga, Japan) following the manufacturer’s protocol. qRT-PCR was conducted using TB Green Premix Ex Taq (Tli RNaseH Plus) and ROX plus (Takara Bio Inc., Otsu, Shiga, Japan). The fold changes in gene expression were calculated upon data normalization. The sequences of the primers are listed in Table S5.

### Western blot

TCMK-1 cells and renal tissues were lysed in radio-immunoprecipitation assay buffer containing phosphatase and protease inhibitors. Total protein concentration was determined using bicinchoninic acid protein assay (Thermo Fisher Scientific, Inc., Waltham, MA, USA) according to the manufacturer’s instructions. Equal amounts of total proteins were loaded into 10% sodium dodecyl sulfate-polyacrylamide gel electrophoresis and transferred onto polyvinylidene difluoride membranes. The membranes were blocked with nonfat milk for 2 h at 37 °C and then incubated with primary antibodies at 4 °C overnight. Subsequently, the membranes were washed in Tris-buffered saline-0.1% Tween-20 and incubated with secondary antibodies at 37 °C for 1.5 h. After incubation, the immunoblot bands were visualized using enhanced chemiluminescence reagent (Beyotime Institute of Biotechnology). Band intensities were quantified by densitometric analysis using ImageJ 1.6.0_20 (National Institutes of Health, Bethesda, MD, USA) after normalized with β-actin (loading control). The details of antibodies are shown in Table S4.

### Co-immunoprecipitation

Briefly, H/R-exposed HEK-293T cells were lysed in sample lysis buffer. Total protein lysates (1 mg) were incubated with 1 mg of antibodies against specific proteins or the indicated rabbit polyclonal IgG control, followed by rotation for 4 h at 4 °C. Next, 20 mL of resuspended volume of Protein A/G Plus-Agarose (Santa Cruz Biotechnology, Dallas, TX, USA) was added to the samples and rotation was continued for 2 h. The resulting complexes were eluted according to the manufacturer’s instructions, and subjected to western blot analysis. The details of antibodies can be found in Table S4.

### Ubiquitylation assay

The ubiquitylation assay was carried out according to a previously described protocol^24^. Briefly, HEK-293T cells were co-transfected with plasmids for *HA-Ub* and the indicated proteins for 48 h, and treated with 10 μM MG132 (Sigma–Aldrich; Merck KGaA, Darmstadt, Germany) for 4 h to inhibit protein degradation. Subsequently, the protein lysates of HEK-293T cells were precipitated and determined by western blot analysis using LC3 and HA antibodies. The details of antibodies are listed in Table S4.

### Statistical analysis

Statistical analyses were performed using PRISM 6 (GraphPad Software, La Jolla, CA). All data were presented as mean ± standard error of the mean (SEM). Survival rates were analyzed by a log-rank test, while other data accorded with normal distribution and homogeneity of variance were compared using Student’s t-test or one-way ANOVA. *P*-values of less than 0.05 were considered statistically significant.

## Supplementary information

Supplementary Information Summary

Supplementary Tables

Supplementary Figure 1

Supplementary Figure 2

Supplementary Figure 3

Supplementary Figure 4

Supplementary Figure 5

Supplementary Figure 6
