# Supplementary material for: Downregulation of XBP1 protects kidney against ischemia-reperfusion injury via suppressing HRD1-mediated NRF2 ubiquitylation
Source: Cell Death Discov. 2021 Mar 2;7:44. doi: 10.1038/s41420-021-00425-z (PMC7925512; doi:10.1038/s41420-021-00425-z)
Supplement: Supplementary file 1 — Supplementary Information Summary [file 41420_2021_425_MOESM1_ESM.docx]

**Supplementary Figure Legends**

**Supplementary Figure. 1:** Genotyping of Xbp1+/- mice. Ef and Kr primers were used to detect FRT flanked region using PCR.

**Supplementary Figure. 2:** In normal TCMK-1 cells, lenti-Xbp1 upregulated the mRNA expression levels of unspliced Xbp1 (Xbp1u) and spliced Xbp1 (Xbp1s), while lenti-shRNA-Xbp1 downregulated the expression levels of these genes.

**Supplementary Figure. 3:** The protein expression levels of XBP1u/s were upregulated by lenti-Xbp1 and downregulated by lenti-shRNA-Xbp1.

**Supplementary Figure. 4:** NRF2 exhibited no significant effects on the expression levels of XBP1 and HRD1.

**Supplementary Figure. 5:** Motif-QSLVPDI (Δ125-131) acted without affecting KEAP1 and GSK-3β

**Supplementary Figure. 6:** HRD1 only binded to the motif-QSLVPDI (Δ125-131), not to other motifs.

**Supplementary Table Legends**

**Supplementary Table. 1:** Primers used for Nrf2 motif deletion mutants.

**Supplementary Table. 2:** Sequences of siRNA and shRNA.

**Supplementary Table. 3:** Details of TCMK-1 cell grouping.

**Supplementary Table. 4:** Details of antibodies used.

**Supplementary Table. 5:** Primer sequences used for qPCR analysis.

**Supplementary Table. 6:** Primer sequences used for PCR genotyping assay.
