# Supplementary material for: Downregulation of XBP1 protects kidney against ischemia-reperfusion injury via suppressing HRD1-mediated NRF2 ubiquitylation
Source: Cell Death Discov. 2021 Mar 2;7:44. doi: 10.1038/s41420-021-00425-z (PMC7925512; doi:10.1038/s41420-021-00425-z)
Supplement: Supplementary file 2 — Supplementary Tables [file 41420_2021_425_MOESM2_ESM.docx]

**Supplementary Tables**

Supplementary Table 1: Primers used for Nrf2 motif deletion mutants.

| Primer | Sequence |
| --- | --- |
| Δ107-111 | 5’- GTACTTTGATGACTGCATGCAGCTTCCGTTTGTAGATGACAATGA-3’  5’- TCATTGTCATCTACAAACGGAAGCTGCATGCAGTCATCAAAGTACA-3’ |
| Δ115-121 | 5’- TTTGGCGCAGACATTCCCGTTTGTAGCTACGTTTCAGTCACTTGTTCCTG-3’  5’- ATCAGGAACAAGTGACTGAAACGTAGCTACAAACGGGAATGTCTGCGCCAA-3’ |
| Δ125-131 | 5’- TGACAATGAGGTTTCTTCGGCTACGTTTCCCGGTCACATCGAGAGCCCAG-3’  5’- CTGGGCTCTCGATGTGACCGGGAAACGTAGCCGAAGAAACCTCATTGTC-3’ |
| Δ139-143 | 5’- ATTCCCGGTCACATCGAGAGCCCAAATCAGGCTCAGTCACCTGAAACTTCT-3’  5’- AGAAGTTTCAGGTGACTGAGCCTGATTTGGGCTCTCGATGTGACCGGGAAT-3’ |

Supplementary Table 2: Sequences of siRNA and shRNA.

| Gene | Sequence |
| --- | --- |
| *Xbp1* | GGTTGAGAACCAGGAGTTAAG |
| *Hrd1* | CGTTCCTGGTACGCCGTCA |

Supplementary Table 3: Details of TCMK-1 cell grouping.

| **Group** | **Definition** |
| --- | --- |
| **XBP1 regulation** |  |
| lenti-*Xbp1* | Cells transduced with lenti-*Xbp1* |
| lenti-shRNA-*Xbp1* | Cells transduced with lenti-shRNA-*Xbp1* |
| lenti-*Xbp1* H/R | H/R-exposed cells transduced with lenti-*Xbp1* |
| lenti-shRNA-*Xbp1* H/R | H/R-exposed cells transduced with lenti-shRNA-*Xbp1* |
| **HRD1 regulation** |  |
| siRNA-*Hrd1* H/R | H/R-exposed cells transfected with siRNA-*Hrd1* |
| plasmid-*Hrd1* H/R | H/R-exposed cells transfected with plasmid-*Hrd1* |
| **NRF2 regulation** |  |
| ML385 H/R | H/R-exposed cells incubated with 5 μM ML385 |
| CDDO-Me H/R | H/R-exposed cells incubated with 0.5 μM CDDO-Me |
| **Control** |  |
| normal control | Cells received no treatment |
| H/R | Cells exposed to H/R |
| lenti-control | Cells transduced with empty lentiviruses |
| lenti-control H/R | H/R-exposed cells transduced with empty lentiviruses |
| siRNA-control H/R | H/R-exposed cells transfected with siRNA-control |
| plasmid-control H/R | H/R-exposed cells transfected with plasmid-control |
| DMSO-control H/R | H/R-exposed cells incubated with 0.1% DMSO |

Supplementary Table 4: Details of antibodies used.

| Antibodies | Source (catalog number) | Dilution |
| --- | --- | --- |
| Primary antibody |  |  |
| Rabbit anti-XBP1 | Abcam (ab37152) | 1:1000 for Western blot |
| Rabbit anti-HRD1 | Abcam (ab170901) | 1:1000 for Western blot |
| Mouse anti-HRD1 | Santa (sc-293484) | 1:200 for immunofluorescence  1:30 for immunoprecipitation |
| Rabbit anti-NRF2 | Cell Signaling Technology (12721) | 1:1000 for Western blot  1:50 for immunoprecipitation |
| Rat anti-NRF2 | Cell Signaling Technology (14596) | 1:400 for immunofluorescence |
| Rabbit anti- HO-1 | Proteintech (10701-1-AP) | 1:1000 for Western blot |
| Mouse anti-β-actin | Proteintech (60008-1-Ig) | 1:5000 for Western blot |
| Mouse anti- HA-Tag | Cell Signaling Technology (2367) | 1:1000 for Western blot |
| Rabbit anti- His-Tag | Cell Signaling Technology (12698) | 1:1000 for Western blot |
| Mouse anti- Flag-Tag | Cell Signaling Technology (8146) | 1:1000 for Western blot  1:50 for immunoprecipitation |
| Rabbit anti-P53 | ABclonal (A3185) | 1:1000 for Western blot  1:200 for immunohistochemistry |
| Rabbit anti-ATM | Proteintech (27156-1-AP) | 1:1000 for Western blot  1:200 for immunohistochemistry |
| Rabbit anti-Caspase 3/p17/p19 | Proteintech (19677-1-AP) | 1:1000 for Western blot |
| IgG Control | Proteintech (30000-0-AP) | 4.0 ug for immunoprecipitation |
| Secondary antibody |  |  |
| Goat anti-rabbit HRP | Proteintech (SA00001-2) | 1:2000 for Western blot |
| Goat anti-mouse HRP | Proteintech (SA00001-1) | 1:2000 for Western blot |
| Goat anti-rabbit HRP | Servicebio (GB23303) | 1:500 for immunohistochemistry |
| Goat anti-rat Cy3 | Servicebio (GB21302) | 1:400 for immunofluorescence |
| Goat anti-mouse Alexa 488 | Servicebio (GB25301) | 1:400 for immunofluorescence |

Supplementary Table 5: Primer sequences used for qPCR analysis.

| Gene | Forward | Reverse |
| --- | --- | --- |
| *Xbp1*u | GTCCATGGGAAGATGTTCTGG | CAGCACTCAGACTATGTGCA |
| *Xbp1*s | AAGAACACGCTTGGGAATGG | CTGCACCTGCTGCGGAC |
| *β-actin* | AGGCCAACCGTGAAAAGATG | TGGCGTGAGGGAGAGCATAG |

Supplementary Table 6: Primer sequences used for PCR genotyping assay.

| Primer Position | Sequence |
| --- | --- |
| Ef | GACGGAATTGGACCCAGAAAGTAGC |
| Er | GGCTTAGCAAGTAAACACGCTTAAAGCTC |
| Kr | CTCCTACATAGTTGGCAGTGTTTGGG |
| L3f | CCCTCGGAGATTAGCCAGGTTG |
| L3r | CACTGACAGCAGAAAGGATACAGAGG |
